# Validation of self-report measures of narcissism against a diagnostic interview

**DOI:** 10.1371/journal.pone.0266540

**Published:** 2022-04-06

**Authors:** Stéphanie Baggio, Katia Iglesias, Miguel Duarte, Rosetta Nicastro, Roland Hasler, Sebastian Euler, Martin Debbané, Vladan Starcevic, Nader Perroud

**Affiliations:** 1 Division of Prison Health, Geneva University Hospitals, Geneva, Switzerland; 2 Institute of Primary Health Care (BIHAM), University of Bern, Bern, Switzerland; 3 School of Health Sciences (HEdS-FR), HES-SO University of Applied Sciences and Arts of Western Switzerland, Fribourg, Switzerland; 4 Service of Psychiatric Specialties, Department of Mental Health and Psychiatry, University Hospitals of Geneva, Geneva, Switzerland; 5 Department of Psychiatry, University of Geneva, Geneva, Switzerland; 6 NCCR Synapsy, Campus Biotech, Geneva, Switzerland; 7 Department of Consultation Psychiatry and Psychosomatics, University Hospital Zurich, Zurich, Switzerland; 8 Faculty of Psychology and Educational Sciences, University of Geneva, Geneva, Switzerland; 9 Developmental Imaging and Psychopathology Lab, Department of Psychiatry, University of Geneva, Geneva, Switzerland; 10 Research Department of Clinical, Educational, and Health Psychology, University College London, London, United Kingdom; 11 University of Sydney, Faculty of Medicine and Health, Sydney Medical School, Nepean Clinical School, Sydney, NSW, Australia; 12 Department of Psychiatry, Dalhousie University, Halifax, Nova Scotia, Canada; University of Padova, ITALY

## Abstract

The Pathological Narcissism Inventory (PNI) and the Narcissistic Personality Inventory (NPI) are often used to screen for pathological narcissism but have rarely been validated against a clinician-administered diagnostic interview. Our study evaluated the convergent validity of the PNI and NPI against a diagnostic interview for narcissistic personality disorder (NPD) in a clinical population. We used data from a psychiatric outpatient center located in Switzerland (n = 123). Correlations between PNI/NPI and NPD ranged between .299 and .498 (common variance 9.0–24.8%). The PNI and NPI should be used carefully to screen for NPD. We highlight a need to increase the compatibility between the conceptual underpinnings of the PNI, NPI and NPD.

## Introduction

Self-report tools are very useful for screening for psychiatric disorders, because they are cost-effective and time-efficient [1, 2]. When needed, administration of self-report instruments is followed by a comprehensive clinical assessment, to ascertain the diagnosis for research or clinical purposes or for health insurance claims. Establishing solid psychometric properties of self-report tools and demonstrating that they possess adequate reliability and validity is paramount for their proper use. However, they should be used cautiously because of limitations such as possible response bias (e.g., socially desirable responding, acquiescent responding, extreme responding), insufficient self-understanding, poor understanding of mental health concepts and issues, cognitive capacity deficits, recall bias [1], and conceptual differences between instruments that purportedly assess the same disorder or construct [3].

Validation of self-report tools for psychiatric disorders is critical and is usually performed in line with guidelines, such as those put forward by the American Psychological Association. According to these guidelines, the validation process includes 1) ascertaining the internal structure of the scale and 2) establishing its convergent and divergent validity via the relationships with conceptually similar and dissimilar constructs, respectively. Self-report screening tools for psychiatric disorders should also be validated against structured or semi-structured diagnostic interviews which assess mental disorders categorically by ascertaining dichotomously whether the disorder is present or not. Unfortunately, this validation is not performed very often because conducting diagnostic interviews is time-consuming and costly. This has been a problem for screening tools used for conditions such as substance use disorders [4], other addictive disorders [5], and depression [6], as well as screening tools used in specific at-risk populations such as individuals with comorbid psychiatric disorders [7], intimate partner violence survivors [8] and older adults [9].

These considerations also apply to self-report screening tools for pathological narcissism: the Pathological Narcissism Inventory (PNI) and the Narcissistic Personality Inventory (NPI). These scales were initially developed to assess narcissism as a personality trait [e.g., 10]. However, some studies considered PNI and NPI to be useful for “pre-screening” of narcissistic personality disorder (NDP) [11] and assessment of pathological variants of narcissism [12] and therefore useful in clinical settings [11, 13]. The psychometric properties of these scales, mostly developed and tested in non-clinical populations, have been well studied, and data on their factor structure, internal consistency, convergent and discriminant validity, and test-retest reliability are available (PNI: [14–16]; NPI: [10, 11, 13, 17, 18]). Overall, these studies concluded that the PNI and NPI were good measures of pathological narcissism, with the expected number of factors (seven factors for the PNI and one or three factors for the NPI, depending on its length), acceptable internal consistency (Cronbach alpha ranging between .57 and .92), good test-retest reliability (correlations ranging between .82 and .85) and acceptable convergent validity with other self-report instruments (maximum correlations ranging between .38 and .62). However, to our knowledge, only one study assessed convergent validity of the NPI using a diagnostic interview for NPD administered by a trained clinical psychologist [12]. This study reported a robust relationship between the NPI and dimensional NPD scores (created by adding the ratings on a 0–2 scale across symptoms), with correlations of .54 in a clinical and .59 in an undergraduate student sample. Considering that these correlations suggest only a partial overlap between the two measures (34.8% of common shared variance), further studies are needed to investigate convergent validity for both the NPI and PNI.

In addition, previous studies did not consider the different dimensions of narcissism, namely grandiosity and vulnerability. These distinct dimensions are implicitly described in the alternative DSM-5 model for personality disorders (section III of the DSM-5), where NPD is conceptualized as consisting of “overt or covert grandiosity”, “variable and vulnerable self-esteem” and “attempts at regulation through attention and approval seeking” [19]. In contrast, the criteria for NPD in Section II of the DSM-5 (“DSM-5 criteria”) only reflect narcissistic grandiosity via symptoms such as arrogance and the sense of self-importance, superiority or entitlement. Narcissistic vulnerability is an aspect of NPD that is less conspicuous and includes a deep sense of personal insufficiency or insecurity, avoidant tendencies and defensiveness. The PNI includes both dimensions, but the NPI does not focus on vulnerability.

Given the paucity of research in this area, lack of clarity as to whether the PNI and NPI could be used as proxies for NPD, and the moderate correlations reported by only one study, this study aimed to further investigate the convergent validity of the PNI and NPI against a diagnostic interview for NPD based on the DSM-5 criteria. We expected a moderate convergent validity. More specifically, we expected that the NPI would have a stronger association with NPD compared to the PNI because the DSM-5 NPD emphasizes narcissistic grandiosity.

## Materials and methods

### Setting

Data were collected between October 2019 and January 2021 by an outpatient center of the Department of Psychiatry of the Geneva University Hospitals that focuses mainly on the diagnosis and treatment of patients suffering from attention deficit hyperactivity disorder (ADHD) and borderline personality disorder. Patients are usually referred for a detailed assessment of these conditions, as well as various forms of psychopathology related to emotion dysregulation.

The inclusion criteria for participation in the present study were as follows: 1) referral for assessment of ADHD, borderline personality disorder or emotion dysregulation, 2) being at least 18 years of age, and 3) providing informed consent for participation in the study and use of health data for research purposes. The study was approved by the Ethics Committee of the Geneva University Hospitals (no. 2021–00694).

### Procedure

Patients were assessed for NPD, ADHD and borderline personality disorder using clinician-administered, semi-structured diagnostic interviews. The SCID-5-PD was administered by RN and NP who were blind to the PNI and NPI scores. In addition, several other psychiatric disorders were clinically assessed: autism spectrum disorder, bipolar disorder, major depressive disorder, anxiety disorders (including generalized anxiety disorder and social phobia), obsessive-compulsive disorder, post-traumatic stress disorder and substance use disorders. Patients also completed NPI and PNI.

### Participants

A total of 264 patients were referred to the center between October 2019 and January 2021. Five (1.9%) patients refused to sign the informed consent and 136 (51.5%) did not complete the NPI and/or PNI and/or were not assessed for NPD. Thus, a total of 123 (47.5%) patients were included in the study. There were no significant sociodemographic and clinical differences between these 123 participants and 136 participants who did not complete narcissism-related measures or were not assessed for NPD.

### Assessment

#### Demographic questionnaire

Sociodemographic variables were collected by a short questionnaire, which included age, gender, marital status (single vs. being in a relationship), level of education (number of years of education), and employment status.

#### Assessment of narcissistic personality disorder

The diagnosis of NPD was made using the Structured Clinical Interview for DSM-IV Personality Disorders, adapted for DSM-5 (SCID-5-PD, [19, 20]). NPD was coded as present or absent, based on the DSM-5 criteria, with a minimum of five out of nine criteria being met.

#### Pathological Narcissism Inventory

The initial PNI consists of 52 items [16]. Here, we used the French version of the short PNI, comprising 28 items rated on a six-point scale (from 0 to 5) [21]. The PNI has been described as a “reliable measure […] of pathological narcissism” [16] with good psychometric properties [14]. From the original seven-factor structures, two second-order factors were derived and assessed via two subscales: the narcissistic grandiosity (12 items) and narcissistic vulnerability (16 items). The internal consistencies in the present sample were 0.85 for the PNI grandiosity and 0.88 for the PNI vulnerability.

#### Narcissistic Personality Inventory

The original NPI consists of 40 items and has been described as providing a “substantial validity evidence” [10]. In this study, we used the French version of the short NPI [18]. Its 13 items are assessed on a yes/no (1/0) scale, with total scores ranging from 0 to 13. The NPI-13 has three subscales: leadership/authority, grandiose exhibitionism and entitlement/exploitativeness. Narcissistic vulnerability is not assessed by the NPI-13. The scale has been described as having a “good validity” and “adequate reliability” [18]. The internal consistency of the NPI in the present sample was 0.74.

#### Assessment of ADHD, borderline personality disorder and other psychiatric disorders

ADHD was assessed based on a semi-structured interview, the ADHD Child Evaluation for Adults (ACE+), which provides a DSM-5-based diagnosis of ADHD [22]. Borderline personality disorder was assessed by means of the SCID-5-PD. Other psychiatric disorders were recorded on the basis of information provided by psychiatrists involved in patients’ care and review of clinical records, including hospitalization reports, discharge summaries and outpatient, follow-up notes. Most of the diagnoses had originally been established via structured or semi-structured clinical interviews conducted by trained clinicians.

### Statistical analyses

A sample size was not computed a priori. We computed a sensitivity power analysis to assess the minimum effect size the study could detect. With n = 123, alpha = .05, power = .80 and a point-biserial correlation, the effect size was d = .25. Therefore, our study could identify small effect sizes.

We first computed descriptive statistics for all variables (percentages for categorical variables and means/standard deviations for continuous variables). Comparisons between participants with and without NPD on all other variables were performed using logistic regressions. We calculated correlations between NPD (based on whether or not the diagnostic criteria for NPD were met and on the number of criteria met), PNI grandiosity, PNI vulnerability and NPI (point-biserial and Spearman correlations for the associations with NPD and Pearson correlations for the associations between PNI and NPI). Normative guidelines about interpretation of correlations provide various criteria, with correlations of .3 [23], .5 [24], and .7 [25, 27] being considered as large. Different types of studies need different sets of empirical guidelines [26]. In our study, we relied on the most stringent criterion of .7 [25, 27] because we examined the correlations between instruments that assess the same construct. All analyses were conducted with Stata 16 and G*Power for the sensitivity power analysis.

## Results

Descriptive statistics and comparisons between participants with and without NPD are reported in Table 1. Participants were on average 34.0 years old and 73.2% were females. About half of them (50.4) were single and currently employed (50.4%). Participants had on average 2.9 psychiatric diagnoses and 11 (8.9%) met the criteria for NPD. Participants with NPD were more likely to be males (p = .039) and to have bipolar disorder (p = .004). The two groups did not differ significantly in terms of other characteristics. Participants with NPD had significantly higher mean scores on all self-report narcissism scales than those without NPD (PNI grandiosity: p < .001, mean difference = 14.4; PNI vulnerability: p = .002, mean difference = 16.1; NPI: p < .001, mean difference = 4.9). Correlations are reported in Table 2. All correlations between self-report narcissism scales and NPD were moderate (< .5), with common variances ranging between 9.0% and 24.8%.

**Table 1:** Descriptive statistics and comparisons between groups.

			Total (n = 123)	Diagnosis of NPD		
				Yes (8.9%, n = 11)	No (91.1%, n = 112)	P^c^
Demographics						
	Age (in years)^a^		34.0 (11.0)	33.2 (9.7)	34.1 (11.2)	.792
	Gender^b^					
		Female	73.2 (90)	45.5 (5)	75.9 (85)	.039
		Male	26.8 (33)	54.5 (6)	24.1 (27)	
	Marital status^b^					
		Single	50.4 (62)	63.6 (7)	49.1 (55)	.642
		In a relationship	49.6 (61)	36.4 (4)	50.9 (57)	
	Level of education (no. of years)^a^		14.9 (3.0)	14.9 (4.0)	14.9 (2.9)	.986
	Employment status^b^					
		Not having a job	49.6 (61)	63.6 (7)	48.2 (54)	.335
		Having a job	50.4 (62)	36.4 (4)	51.8 (58)	
Psychiatric disorders						
	ADHD^b^		67.5 (83)	63.6 (7)	67.9 (76)	.776
	Autism spectrum disorder^b^		0.0 (0)	0.0 (0)	0.0 (0)	-
	Bipolar disorder^b^		13.8 (17)	45.5 (5)	10.7 (12)	.004
	Borderline personality disorder^b^		62.6 (77)	72.7 (8)	61.6 (69)	.471
	Major depressive disorder^b^		57.7 (71)	36.4 (4)	59.8 (67)	.144
	Social anxiety disorder^b^		27.6 (34)	45.5 (5)	25.9 (29)	.176
	Substance use disorder^b^		62.6 (77)	54.6 (6)	63.4 (71)	.564
	No. of disorders^a^		2.9 (1.1)	3.2 (0.9)	2.9 (1.1)	.396
Self-report narcissism scales						
	PNI grandiosity^a^		27.5 (11.9)	40.6 (10.4)	26.2 (11.3)	.001
	PNI vulnerability^a^		36.5 (15.4)	51.1 (18.0)	35.0 (14.4)	.002
	NPI^a^		3.5 (2.8)	8.0 (2.7)	3.1 (2.4)	< .001

NPD: narcissistic personality disorder; PNI: Pathological Narcissism Inventory; NPI: Narcissistic Personality Inventory

^a^ Means and standard deviations are given

^b^ Percentages and n are given.

^c^ p-values for logistic regressions are reported.

**Table 2:** Correlations between narcissism measures.

	PNI grandiosity	PNI vulnerability	NPI
Binary NPD (based on whether or not diagnostic criteria have been met)^a^	.347	.299	.498
No. of NPD criteria met^b^	.451	.353	.453
PNI grandiosity^c^	-	.816	.509
PNI vulnerability^c^	-	-	.417

NPD: narcissistic personality disorder; PNI: Pathological Narcissism Inventory; NPI: Narcissistic Personality Inventory

^a^ Point-biserial correlations.

^b^ Spearman correlations.

^c^ Pearson correlations.

## Discussion

This study aimed to evaluate in a sample of outpatients the convergent validity of the PNI and NPI, the most commonly used self-report scales for pathological narcissism, against a diagnostic interview for NPD.

Our main finding is that the PNI and NPI were moderately associated with a measure of NPD, with the correlations ranging between .299 and .498 and therefore indicating a maximum of 24.8% of shared variance. This is in line with the results of another study making similar comparisons between self-report scales and clinician-rated measures of personality pathology [28].

A previous study reported a correlation of .59 between the total NPI score and a three-point measure of DSM-IV NPD and concluded that the NPI and NPD were “strongly” related, with NPI therefore being “quite relevant” for assessment of NPD [12]. A correlation of >.5 is a commonly used criterion for convergent validity, but it means that the instruments only share a quarter of common variance, which is insufficient to conclude that they measure the same construct. Previous studies suggested a correlation of >.7 as a more stringent criterion for establishing adequate convergent validity [25, 27]. A correlation of .7 means that the instruments share almost a half of common variance. If the screening self-report instrument assesses the same construct as the diagnostic interview and if both have the same or similar theoretical foundations, the correlation between them should be high for the screening self-report instrument to be considered to have a good convergent validity. In line with Hemphill [26], we suggest that different guidelines should be applied to different types of studies/areas of investigations. When researchers examine the correlations between different measures of the same construct, a more stringent criterion may be desirable. Further studies, such as simulation studies, should test whether such a criterion can be sufficiently justified.

The NPI, which does not assess narcissistic vulnerability, had the strongest correlation with NPD (r = .498) and the PNI narcissistic grandiosity displayed a better criterion validity than the PNI narcissistic vulnerability (r = .347 and r = .299, respectively). A possible explanation for a weaker performance of the PNI narcissistic vulnerability relates to the fact that the diagnosis of NPD encompasses grandiosity, entitlement, lack of empathy and tendency to be exploitative, without capturing specific narcissistic vulnerabilities. This calls for efforts to “harmonize” theoretical approaches to NPD [29, 30], make NPD more compatible with what self-report instruments such as PNI and NPI assess, and capture the complexity of the narcissism construct [31]. This has already been attempted with the DSM-5 alternative model for personality disorders and the next step would be to investigate the convergent validity of the NPI and PNI against a structured clinical interview based on this model, e.g., the Structured Clinical Interview for the DSM-5 Alternative Model for Personality Disorders (SCID-5-AMPD) [32].

This study has several limitations. First, its sample size was modest, which might have led to a reduced number of patients with NPD (n = 11). Therefore, our findings should be replicated in studies with larger samples. Second, the study was conducted in outpatients with various psychiatric conditions and its findings are not necessarily generalizable to the general population. Indeed, psychometric properties of self-report scales are often weaker in samples with comorbid disorders than in the general population or samples with a single psychiatric disorder [7, 33, 34]. In the present study, a large proportion of patients (>60% in the total sample and in the sample of individuals diagnosed with NPD) had a co-occurring borderline personality disorder and ADHD. It is conceivable that these co-occurring conditions affected the scores on the PNI and NPI, although such a possibility has not been thoroughly investigated. The mean scores on the PNI vulnerability were higher than on the PNI grandiosity, which could be a consequence of the co-occurring disorders. Thus, studies showed that narcissistic vulnerability and self-esteem dysregulation were prominent among people with borderline personality disorder [35, 36]. It could have led to higher scores on PNI vulnerability, which includes a lower-order factor of contingent self-esteem. Future studies in larger samples from the general population are needed to further investigate the psychometric properties of the PNI and NPI. A third limitation was that individuals with NPD do not have a well-developed capacity for self-reflection [37, 38]. This could have reduced the correlation between self-reported questionnaires and the clinician-administered assessment of NPD. Finally, we did not control for inter-rater reliability. However, an excellent inter-rater reliability has been reported for the SCID I and II [39].

Overall, our findings suggest that the PNI and NPI should be carefully used to screen for NPD. Researchers and clinicians should bear in mind that these instruments were originally developed to screen for narcissism as a personality trait and were not designed as a screening tool for NPD. This study also shed light on the inconsistencies between the different concepts of narcissism, calling once more for a consensus on this topic.

## Supporting information

S1 Dataset (XLSX)
